# Corticosteroid and antiviral therapy for Bell's palsy: A network meta-analysis

**DOI:** 10.1186/1471-2377-11-1

**Published:** 2011-01-05

**Authors:** Pawin Numthavaj, Ammarin Thakkinstian, Charungthai Dejthevaporn, John Attia

**Affiliations:** 1 Section for Clinical Epidemiology and Biostatistics, Faculty of Medicine, Ramathibodi Hospital, Mahidol University, Bangkok, Thailand; 2 Division of Neurology, Department of Medicine, Faculty of Medicine, Ramathibodi Hospital, Mahidol University, Bangkok, Thailand; 3 Centre for Clinical Epidemiology and Biostatistics, The University of Newcastle, Hunter Medical Research Institute, and Department of General Medicine, John Hunter Hospital, Newcastle, NSW, Australia

## Abstract

**Background:**

Previous meta-analyses of treatments for Bell's palsy are still inconclusive due to different comparators, insufficient data, and lack of power. We therefore conducted a network meta-analysis combining direct and indirect comparisons for assessing efficacy of steroids and antiviral treatment (AVT) at 3 and 6 months.

**Methods:**

We searched Medline and EMBASE until September 2010 using PubMed and Elsviere search engines. A network meta-analysis was performed to assess disease recovery using a mixed effects hierarchical model. Goodness of fit of the model was assessed, and the pooled odds ratio (OR) and 95% confidence interval (CI) were estimated.

**Results:**

Six studies (total n = 1805)were eligible and contributed to the network meta-analysis. The pooled ORs for resolution at 3 months were 1.24 (95% CI: 0.79 - 1.94) for Acyclovir plus Prednisolone and 1.02 (95% CI: 0.73 - 1.42) for Valacyclovir plus Prednisolone, versus Prednisolone alone. Either Acyclovir or Valacyclovir singly had significantly lower efficacy than Prednisolone alone, i.e., ORs were 0·44 (95% CI: 0·28 - 0·68) and 0·60 (95% CI: 0·42 - 0·87), respectively. Neither of the antiviral agents was significantly different compared with placebo, with a pooled OR of 1·25 (95% CI: 0·78 - 1·98) for Acyclovir and 0·91 (95% CI: 0·63 - 1·31) for Valacyclovir. Overall, Prednisolone-based treatment increased the chance of recovery 2-fold (95% CI: 1·55 - 2·42) compared to non-Prednisolone-based treatment. To gain 1 extra recovery, 6 and 26 patients need to be treated with Acyclovir and prednisolone compared to placebo and prednisolone alone, respectively.

**Conclusions:**

Our evidence suggests that the current practice of treating Bell's palsy with AVT plus corticosteroid may lead to slightly higher recovery rates compared to treating with prednisone alone but this does not quite reach statistical significance; prednisone remains the best evidence-based treatment.

## Background

Bell's palsy is a condition characterized by an acute onset of facial nerve palsy with no known cause. The incidence is about 20/year/100,000 population[1], and leads to a considerable disturbance in social activities among patients[2].

Although the actual cause of Bell's palsy is unknown, the widely accepted mechanism is inflammation of the facial nerve during its course through the bony labyrinthine part of the facial canal, which leads to compression and demyelination of the axons, and disruption of blood supply to the nerve itself[3]. Previous studies have suggested viral infection as the etiology of the disease based on serological evidence;[4,5] for example, positive serology for Herpes Simplex virus (HSV) has been reported in 20-79% of patients.

Treatment of Bell's palsy varies, and no clear consensus exists. Most physicians prescribe corticosteroids as a primary treatment due to its potential to reduce swelling and inflammation. The addition of antiviral treatment (AVT) such as Acyclovir or Valacyclovir is aimed at eradication of HSV infection. Acyclovir, a nucleoside analog, inhibits HSV replication through inhibition of viral DNA polymerase. It is absorbed slowly from the gastrointestinal tract, necessitating the use of a five-times daily regimen. Valacyclovir, a Valine derivative of Acyclovir, is claimed to lead to higher drug levels through conversion to Acyclovir by intestinal and hepatic esterase enzymes, leading to less intensive regimens. Its distribution, cellular kinetics, mechanism, and excretion are otherwise identical to Acyclovir[6].

Efficacy of AVT in Bell's palsy is still not established, and the question exists whether adding AVT to another treatment such as corticosteroid can lead to better and faster recovery compared with corticosteroids alone or without treatment. The original Cochrane systematic review of this topic[7] included 3 studies; these were heterogeneous however and could not be pooled. Since that date, there have been at least 3 more large individual studies[8-13] and 3 more recent meta-analyses [14-16] published, although the comparator groups vary considerably and make traditional direct meta-analysis difficult. The meta-analsis by de Almeida et al. applied logistic regression analysis to assess interation effects between corticosteroids and AVT, however this does not account for heterogeneity and did not estimate the individual effect of AVT(Acyclovir or Valacyclovir). Furthermore, the review combined adult and paediatric studies (6 studies), in which dosages and effects of treatments may be different. The most recent update of the Cochrane systematic review included 6 studies [17], but did not look at the effects of AVTs alone. We therefore conducted a systematic review and network meta-analysis with the aim of comparing complete recovery rates at 3 and 6 months for corticosteroids, AVT (Acyclovir or Valacyclovir), or the combination of both for treatment of adult Bell's palsy. Performing a network meta-analysis by borrowing information from indirect comparisons integrates information about relative treatment efficacy[18-20]. This technique has been applied in systematic reviews of other clinical topics such as chronic insomnia[21], polymer-based drug-eluting stents[22], and highly-active AVT[23].

## Methods

### Search strategy

One author (NP) located studies in MEDLINE (from 1966 to August 2010) and EMBASE (from 1950 to September 2010) using PubMed and Ovid search engines. Search terms used were as follows: (Bell's palsy or idiopathic facial palsy) and (antiviral agents or acyclovir or valacyclovir), limited to randomized controlled trials. Search strategies for both databases are described in the additional file 1.

### Selection of study and inclusion/exclusion criteria

Abstracts and/or full papers of identified studies were reviewed by one author (NP) and checked by another author (TA). Studies were included if they were RCTs, and studied subjects aged 18 years or older with sufficient data. Non-English papers were excluded from the review. Where eligible papers had insufficient information, corresponding authors were contacted by e-mail for additional information. The reference lists of the retrieved papers were also reviewed to identify relevant publications. Where there were multiple publications from the same study group, the most complete and recent results were used.

### Data extraction & Quality assessment (QA)

Data extraction was independently performed in duplicate by PN and AT using a standardized data extraction form, which included study design, sample size, patient characteristics (i.e., age, gender), type of intervention and comparator, outcomes, and follow-up time. Any disagreement was resolved by discussion.

Quality of studies was also independently assessed by PN and AT based on a modified Jadad score which takes into account randomization technique, allocation concealment, blinding, intention to treat, and patient attrition[24]. Each item was graded 2, 1, or 0 for appropriately, partially, and inappropriately described methods. Any disagreement between the two reviewers was discussed and resolved by consensus.

### Outcome

Complete recovery was defined as a score ≤2 on the House-Brackman Facial Recovery scale[12,13,25], ≥ 8 on the Facial Palsy Recovery Index[8], > 36 points on theYanagihara score[10], or 100 on the Sunnybrook scale[9].

### Statistical analysis

For direct meta-analysis, the odds ratio (OR) and variance of each study were estimated and pooled. Heterogeneity of ORs was assessed using Cochran's Q test and I^2^. If heterogeneity was present, ORs were pooled using the random effects model, i.e. Der-Simonian and Laird method. Meta-regression was applied to assess whether age, gender, and QA were sources of heterogeneity, if these data were available. Contour enhanced funnel plots were used to detect publication bias due to small study effects [26-29]. Trim and fill meta-analysis was applied to impute the number of missing studies [30].

For network meta-analysis, treatment groups were considered in a mixed effects hierarchical model with logit link function using the xtmelogit command in STATA[31]. The treatments were included in the model as fixed effects whereas the study was included as a random effect. Likelihood estimates were used for estimation of parameters in the model. Goodness of fit of the model was assessed using the Hosmer-Lemeshow Chi-square test. The pooled ORs and 95% confidence intervals (CI) were estimated by exponential coefficients of treatments. Discrepancy of treatment effects between direct and indirect meta-results was then assessed using the standardized normal method (Z), i.e. by dividing the difference by its standard error[20,32]. Number needed to treat/harm (NNT/NNH) was estimated using probability of complete recovery and ORs derived from the mixed effects hierarchical model, where the ORs were converted to risk ratios following the method of Zhang et al[33]. All analyses were performed using STATA version 11.0. P values < 0·05 were considered as statistically significant except for the heterogeneity test where <0·10 was used.

## Results

**Figure 1:**
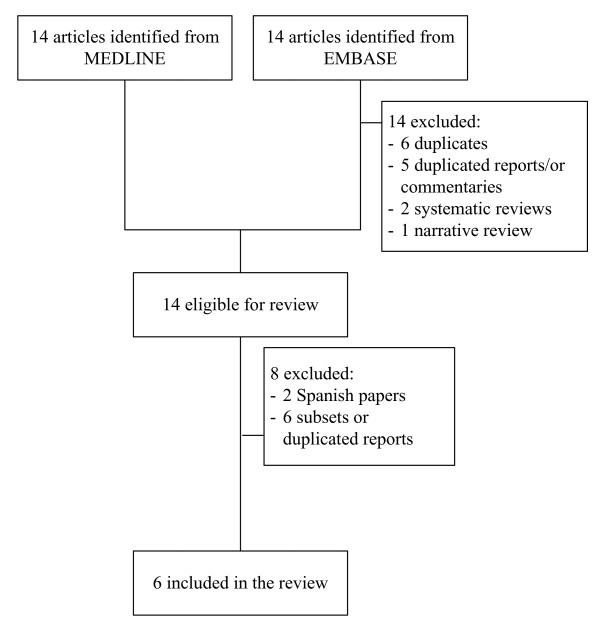
**Diagram of selection of studies**.

A flow diagram of study selection is shown in Figure 1. Fourteen and twenty-five studies were identified from MEDLINE and EMBASE databases, respectively. Among these 39 studies, 1 studies were duplicates, 13 studies were ineligible, leaving 12 studies to review. Six studies were excluded; two were in Spanish, one was in German, and 3 were subsets or duplicated reports, leaving six studies[8-10,12,13,25] with a total of 1805 patients for analysis. Characteristics of the 6 included studies are described in table 1. Five studies[8-10,12,13] compared recovery rates of AVT plus corticosteroid with corticosteroid alone or placebo; the remaining study directly compared the recovery rate of AVT against corticosteroid[25]. Two studies were based on factorial design[9,12] and the others were parallel randomized control trials. The AVTs used were Acyclovir for 4 studies[8,12,13,25] and Valacyclovir for the other 2 studies[9,10]. Prednisolone was the major corticosteroid used. Mean age of participants in these studies ranged from 40 to 50 years, and percentage of male participants ranged from 45% to 59%. Median quality assessment score was 8 (range = 2-12).

**Table 1:** Baseline characteristics of included studies

Author (Year)	N	Mean age	% Male Participants	Mean disease severity score	Type of treatment		Outcomes	FU time(month)	QA score
								
					Intervention (dosage)	Control			
Adour et al. (1996) [8]	99	43	50	3 (FPRP)	Acyclovir 2,000 mg/day × 10 days Prednisolone 30 mg/kg/day × 5 days, 10 mg/day × next 5 days	Prednisolone with the same dosage	FPRI ≥ 8	4	7
De Diego et al. (1998)[25]	101	-	-	-	Acyclovir 2,400 mg/day ×10 days	Prednisolone 1 mg/kg/day × 10 days, taper over next 6 days	HB grade ≤ II, FPRI score≥ 8	3	3
Hato et al. (2007)[10]	221	50	53	15 (Yanagihara)	Valacyclovir 1,000 mg/day × 5 days Prednisolone 60 mg/day for 5 days, taper with Mecobalamin 1·5 mg/day × 6 months	Placebo	Yanagihara score > 36 points, no facial contracture or synkinesis	6	9
Sullivan et al. (2007) [12]	551	44	51	3.6 (HB)	Acyclovir 2000 mg/day × 10 days Prednisolone 50 mg/day × 10 days	Placebo	HB grade I	3, 9	12
Yeo et al. (2008)[13]	91	41	45	3.7 (HB)	Acyclovir 2,400 mg/day × 5 days Prednisolone 1 mg/kg/day × 4 days with maximum of 80 mg/day, 60 mg/day × 5-6 day, 40 mg/day × 7-8 day, 20 mg/day × 9-10 day	Placebo	HB grade ≤ II	2, 6	2
Engstrom et al. (2008) [9]	829	40	59	4 by HB	Valacyclovir 3,000 mg/day × 7 days Prednisolone 60 mg/day × 5 days, 10 mg/day until 10 days	Placebo	Sunnybrook 100/100, HB ≤ II	1, 2, 3, 6, 12	12

### Direct comparisons

Among 6 studies, 3 studies[8,12,13] compared recovery rates within 3 months between Acyclovir plus Prednisolone versus Prednisolone alone (table 2). Pooled treatment effects were heterogeneous (Chi-sqaure = 6·30 (d.f. = 2) p = 0·043; I^2^= 68.3%). Using the random effects model gave a pooled OR of 1·39 (95% CI: = 0·52 - 3·75), i.e. patients who received Acyclovir plus Prednisolone were about 40% more likely to recover within 3 month than patients who received Prednisolone alone, although this did not reach statistical significance. Only 2 studies[9,10] compared recovery rates between Valacyclovir plus Prednisolone and Prednisolone. They were homogeneous and the pooled OR with fixed effect model was 1·17 (95% CI: 0·75 - 1·81).

**Table 2:** Describe numbers of recovery according to treatment groups for each included study

Author	Treatment groups	Recovery at 3 months	Total N	Recovery after 3 months	Total N
Adour et al. [8]	Acy+Pred	34	38	49	53
	Pred	20	29	35	46
De Diego[25]	Acy	42	54	-	-
	Pred	44	47	-	-
Engstorm et al. [9]	Val+Pred	134	206	149	206
	Val	113	207	120	207
	Pred	137	210	150	210
	Plac	111	206	127	206
Hato et al. [10]	Val+Pred	94	114	110	114
	Pred	80	107	96	107
Sullivan et al. [12]	Acy+Pred	99	124	115	124
	Acy	76	123	96	123
	Pred	109	127	122	127
	Plac	79	122	104	122
Yeo et al. [13]	Acy + Pred	36	44	41	44
	Pred	35	47	40	47

**Figure 2:**
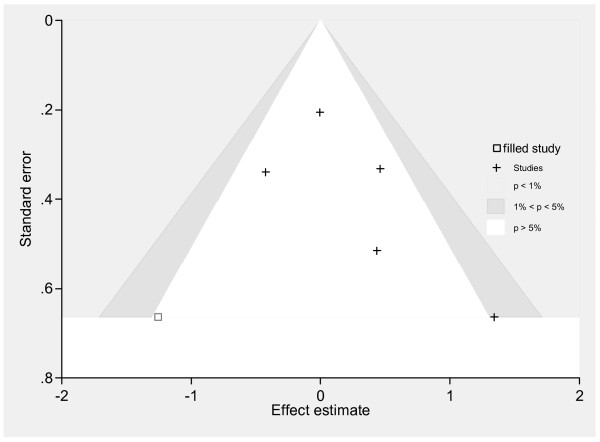
**Contour enhanced funnel plot: AT plus Prednisolone versus Prednisolone**.

Combining 5 studies[8-10,12,13] to assess the effect of AVT (Acyclovir/Valacyclovir) plus Prednisolone versus Prednisolone found moderate heterogeneity (Chi-sqaure = 7.78 (d.f. = 4) p = 0.100; I^2 ^= 48.6%). The pooled OR with a random effect model was 1.21 (95% CI: 0.77 - 1.89), i.e. AVT plus Prednisolone had 21% higher chance of complete recovery than Prednisolone alone but this was non-significant. Meta-regression suggested that quality assessment score and disease severity at baseline might be a source of heterogeneity, reducing I^2 ^from 48.6% to 32.5% and 15.5% respectively although both variables were non-significantly associated with treatment effects. Pooling studies with quality assessment scores > 8 (median) suggested no benefit of AVT plus Prednisolone compared with Prednisolone alone (pooled OR = 1.01, 95% CI: 0.74 - 1.37). Two studies[8,10] had more severe patients at baseline (i.e., mean scores were 3 by facial palsy recovery index and 15 by Yanagihara score) compared with the other 3 studies[9,12,13] (i.e., mean scores were 3.6 to 4 by HB), see table 1. Pooling within the severe group only suggested the possibility of a treatment effect but this was non-significant (OR = 2.04, 95% CI: 0.93 - 4.46) while treatment effect was close to the null in the less severe group (OR = 0.94, 95%CI: 0.66 - 1.33). Contour enhanced funnel plots suggested asymmetry, i.e., four studies lay in the non-sigificant area (white, p > 5%) but the study with the largest treatment effect and SE lay in the positive-moderate significant area (1% ≤ p ≤ 5%, see Figure 2), suggesting that the asymmetry might be due to missing, non-significant studies. "metatrim" analysis indicated that only one negative study with borderline significance was missing; adjusting for this presumed missing studies resulted in an ORof 1.08 (95% CI: 0.66 - 1.76).

Three studies[8,12,13] reported the recovery rate between direct comparisons of Acyclovir plus Prednisolone versus Prednisolone after receiving treatments at 4 to 9 months (table 2). Treatment effects were statistically heterogeneous (Chi-square = 6·07 (d.f. = 2), p = 0·048; I2 = 67·0%), and the random effects pooled OR was 1·63 (95% CI: 0·47 - 5·75). Pooling effects of Valacyclovir plus Prednisolone versus Prednisolone was performed based on only 2 heterogeneous studies[9,10]. The random effects pooled OR was 1·58 (95% CI: 0·55 - 4·48). There was only 1 study each that compared Acyclovir[12] or Valacyclovir[9] alone versus Prednisolone and this was insufficient for pooling.

### Network meta-analysis

All six studies[8-10,12,13,25] were able to contribute to a network meta-analysis, for a total of 1,805 patients, as described in table 2. Overall, 177 (9·8%), 207 (11·5%), 567 (31·4%), and 328 (18·2%) received only Acyclovir, Valacyclovir, Prednisolone, or placebo whereas 206 (11·4%) and 320 (17·7%) received Acyclovir plus Prednisolone and Valacyclovir plus Prednisolone, respectively. One-thousand two-hundred and forty-three patients had complete recovery (68·9%) within three months.

**Figure 3:**
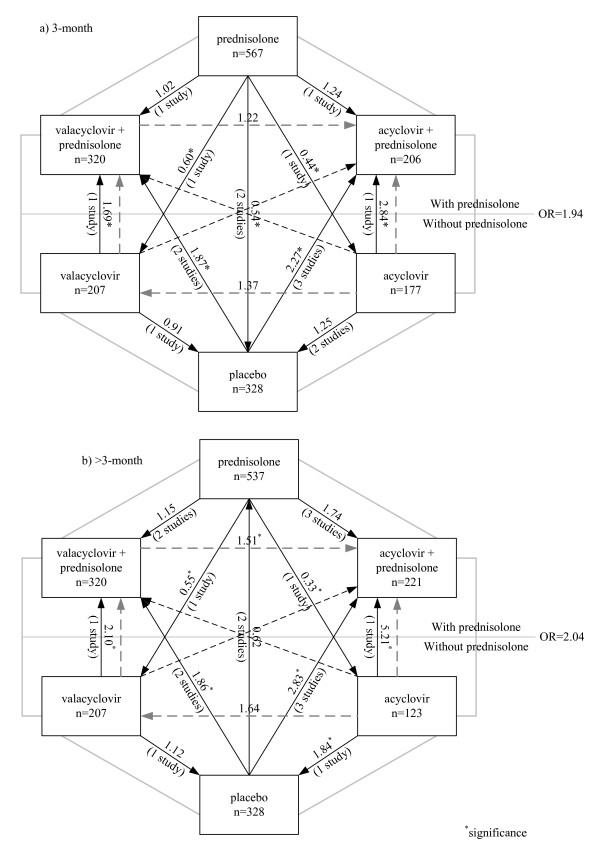
**Comparisons of recovery rates between treatment groups: A network meta-analysis**. a) at 3 months. b) at > 3 months

All treatment comparisons and results of the network meta-analysis for 3 month outcomes are displayed in Figure 3a. Each line in the figure represents a randomized comparison, in which the head and the tail of that line refer to intervention and reference groups respectively. Figures on the lines refer to estimated indirect ORs for that comparison; ORs less than one indicate that the intervention group had lower recovery than the reference group, whereas ORs higher than one mean that the intervention group had higher recovery than the reference group. The mixed-effects model with a random intercept and a constant slope was applied to assess treatment effects. The estimated variance between studies was 0·17 (95% CI: 0·04 - 0·76). Hosmer-Lemeshow test found that the model was a good fit (Chi-square = 12·99, p = 0·072).

Treatment effects for all comparisons are shown in Figure 3a and table 3. Adding Acyclovir or Valacyclovir to Prednisolone did not significantly increase recovery rate when compared with Prednisolone alone, although the point estimates suggest a potential benefit of the combination, i.e., ORs were 1·24 (95% CI: 0·79 - 1·94) and 1·02 (95% CI: 0·73 - 1·42) for Acyclovir and Valacyclovir, respectively. For monotherapy, either Acyclovir (OR = 0·44, 95% CI: 0·28 - 0·68) or Valacyclovir (OR = 0·60, 95% CI: 0·42 - 0·87) led to significantly lower recovery than Prednisolone alone and this was not significantly different compared with placebo (OR = 1·25, 95% CI: 0·78 - 1·98 for Placebo versus Acyclovir; OR = 0·91, 95% CI: 0·63 - 1·31 for Placebo versus Valacyclovir). These data indicate that Prednisolone led to higher recovery rates than not receiving Predisolone. We therefore combined all Prednisolone groups (regardless of additional AVT) compared with non-Prednisolone (i.e., Acyclovir, Valacyclovir, and Placebo). The estimated OR was 1·94 (95% CI: 1·55 - 2·42), i.e., Prednisolone-based treatment increase the chance of recovery by 3 months by 2 fold compared to non-Prednisolone-based treatment.

**Table 3:** Comparisons of treatment effects on disease recovery at 3 months between direct and network meta-analyses

Time at assessment	Intervention Group	Reference Group	Direct meta-analysis				Network meta-analysis				Discrepancy	
			
			n	OR	95% CI	SE	n	OR	95% CI	SE	Z	P value
3 month	Acy + Pred	Pred	409	1·39	0·52-3·75	0·32	773	1·24	0·79-1·94	0·28	0.27	0.788
	Val + Pred	Pred	637	1·17	0·75-1·81	0·22	887	1·02	0·73-1·42	0·17	0·49	0·621
	Acy	Pred	351	0·26	0·15-0·46	0·28	744	0·44	0·28-0·68	0·10	-1·77	0·077
	Val	Pred	417	0·64	0·43-0·95	0·21	774	0·60	0·42-0·87	0·11	0·27	0·785
	Plac	Pred	665	0·50	0·36-0·70	0·17	895	0·54	0·40-0·74	0·09	-0·40	0·689
	Acy+Pred	Acy	247	2·45	1·33 - 4·53	0·29	383	2·84	1·68 - 4·78	0·75	0·83	0·20
	Val+Pred	Acy	-	-	-	-	497	2·32	1·37 - 3·93	0·62	-	-
	Val	Acy	-	-	-	-	384	1·37	0·79 - 2·37	0·38	-	-
	Plac	Acy	245	1·14	0·65 - 1·98	0·27	505	1·25	0·78 - 1·98	0·29	-0·23	0·41
	Acy+Pred	Val	-	-	-	-	413	2·06	1·20 - 3·56	0·57		
	Val+Pred	Val	413	1·55	1·02 - 2·35	0·20	527	1·69	1·16 - 2·48	0·33	-0·22	0·411
	Plac	Val	413	0·97	0·65 - 1·45	0·20	535	0·91	0·63 - 1·31	0·17	0·24	0·404
	Acy+Pred	Plac	246	2·16	1·17 - 4·00	0·29	534	2·27	1·31 - 2·66	0·55	-0·08	0·468
	Val+Pred	Plac	412	1·59	1·05 - 2·42	0·20	648	1·87	1·31 - 2·66	0·34	-0·41	0·340
	Acy+Pred	Val+Pred	-	-	-	-	526	1·22	0·72 - 2·06	0·32	-	-

6 month	Acy + Pred	Pred	441	1·63	0·47 - 5·75	0·83	758	1·74	0·93-3·24	0·56	-0·07	0·474
	Val + Pred	Pred	637	1·58	0·55 - 4·48	0·40	857	1·15	0·78-1·69	0·23	0·67	0·246
	Acy	Pred	250	0·15	0·04 - 0·41	0·25	660	0·33	0·18-0·61	0·10	-2·93	0·002
	Val	Pred	417	0·55	0·36 - 0·85	0·21	744	0·55	0·37-0·81	0·11	0·00	0·500
	Plac	Pred	665	0·44	0·17 - 1·14	0·34	865	0·62	0·43-0·88	0·11	-0·96	0·169
	Acy+Pred	Acy	247	3·6	1·54 - 9·07	0·41	344	5·21	2·48 - 10·94	1·97	-0·18	0·427
	Val+Pred	Acy	-	-	-	-	443	3·45	1·78 - 6·68	1·16	-	-
	Val	Acy	-	-	-	-	330	1·64	0·85 - 3·18	0·55	-	-
	Plac	Acy	245	1·63	0. 80 - 3·34	0·34	451	1·84	1·02 - 3·33	0·56	-0·18	0·427
	Acy+Pred	Val	-	-	-	-	428	3·17	1·57 - 6·40	1·14		
	Val+Pred	Val	413	1·89	1·23 - 2·92	0·21	527	2·10	1·40 - 3·15	0·44	-0·22	0·414
	Plac	Val	413	1·16	0·77 - 1·76	0·20	535	1·12	0·76 - 1·65	0·22	0·12	0·453
	Acy+Pred	Plac	246	2·21	0·89 - 5·82	0·43	549	2·83	1·46 - 5·47	0·95	-0·24	0·406
	Val+Pred	Plac	412	1·63	1·05 - 2·51	0·21	648	1·86	1·26 - 2·77	0·38	-0·30	0·381
	Acy+Pred	Val+Pred	-	-	-	-	541	1·51	0·75 - 3·05	0·54	-	-

These effects were largely consistent when recovery was judged at later time points (Figure 3b and table 3). Acyclovir (OR = 1·74, 95% CI: 0·93 - 3·24) or Valacyclovir (OR = 1·15, 95% CI: 0·78 - 1·69) added to Prednisolone tended to improve recovery rates compared to receiving Prednisolone alone but the effects were non-significant. Mono-therapy with either Acyclovir (OR = 0·33, 95% CI: 0·18 - 0·68) or Valacyclovir (OR = 0·55, 95% CI: 0·37 - 0·81), or placebo (OR = 0·62, 95% CI: 0·43 - 0·88) led to significantly lower recovery than Prednisolone mono-therapy. Compared with Acyclovir, Acyclovir or Valacyclovir plus Prednisolone was more beneficial than Acyclovir alone, with ORs of 5·21 (95% CI: 2·48 - 10·94) and 3·45 (95% CI: 1·78 - 6·68) respectively; trends were similar for Valacyclovir. Overall, the effect of prednisolone-based regimens compared to those without prednisolone was similar to that at 3 months, with OR of 2·04 (95% CI 1·57 - 2·64).

### Comparison of direct and network meta-analysis

Results of direct and network analyses are compared in the last column of table 3. Direction of treatment effects between both methods were similar for all comparisons, although the direct approach provided higher treatment effects in both positive and negative directions. The degree of discrepancy between the 2 methods was very small except for Acyclovir versus Prednisolone, where Z was large and significant at 3 and 6 months (1·77, p = 0·077; and -2·93, p = 0·002 respectively). Precision of treatment effects was generally higher in the network meta-analysis than the direct one.

Finally, estimates of NNT and NNH for recovery at 3 and 6 months are given in table 4. We found that 6 patients need to be treated with Acyclovir and prednisolone compared to placebo in order to gain 1 extra recovery and this had a tight confidence interval. By contrast, the benefit of combination AT and prednisolone compared to prednisolone alone was less certain, with 26 patients needing to be treated with Acyclovir plus predinisolone in order to gain 1 extra patient with recovery at 3 months, and this had wide confidence intervals.

**Table 4:** Estimated numbers needed to treat and numbers needed to harm of treatments

Intervention Group	Reference Group	NNT/NNH (3 months)			NNT/NNH (6 months)		
		Point Estimate	95% CI		Point Estimate	95% CI	
Acy + Pred	Pred	NNT 26	NNH 21	NNT 10	NNT 15	NNH 93	NNT 9
Val + Pred	Pred	NNT 271	NNH 16	NNT 17	NNT 52	NNH 26	NNT 16
Acy	Pred	NNH 6	NNH 3	NNH 13	NNH 5	NNH 3	NNH 12
Val	Pred	NNH 9	NNH 5	NNH 37	NNH 10	NNH 5	NNH 31
Plac	Pred	NNH 8	NNH 5	NNH 17	NNH 13	NNH 6	NNH 52
Acy+Pred	Acy	NNT 8	NNT 10	NNT 4	NNT 6	NNT 8	NNT 5
Val+Pred	Acy	NNT 3	NNT 15	NNT 5	NNT 7	NNT 12	NNT 6
Val	Acy	NNT 15	NNH 18	NNT 6	NNT 14	NNH 34	NNT 7
Plac	Acy	NNT 21	NNH 17	NNT 8	NNT 11	NNT 296	NNT 7
Acy+Pred	Val	NNT 6	NNT 22	NNT 4	NNT 4	NNT 10	NNT 3
Val+Pred	Val	NNT 8	NNT 27	NNT 5	NNT 6	NNT 13	NNT 4
Plac	Val	NNH 43	NNH 9	NNT 15	NNT 37	NNH 15	NNT 9
Acy+Pred	Plac	NNT 6	NNT 16	NNT 5	NNT 6	NNT 14	NNT 4
Val+Pred	Plac	NNT 7	NNT 16	NNT 5	NNT 9	NNT 22	NNT 6
Acy+Pred	Val+Pred	NNT 26	NNH 14	NNT 8	NNT 18	NNH 21	NNT 8

## Discussion

Our network meta-analysis indicates that treatment with AVT alone or placebo is significantly inferior to Prednisolone alone; the effect of AVT alone and placebo are similar to each other. Current practice of adding AVT (either Acyclovir or Valacyclovir) in the regimen with Prednisolone may increase disease recovery rates compared with Prednisolone alone, but at this point this difference is not statistically significant. Prednisolone remains the strongest evidence-based treatment, whether compared to placebo or AVT monotherapy.

The possible explanations for the lack of any incremental effect of AVT when added to corticosteroids might include:

• corticosteroids reduces the inflammatory process in Bell's palsy and this facilitates remyelination of facial nerve.

• Bell's palsy is a post-infectious immune mediated facial neuropathy rather than direct viral infection

• There may be a small incremental increase in efficacy but there is not sufficient power, even with all the trials to date, to demonstrate this. Large RCTs are needed to specifically compare corticosteroid and corticosteroid plus AVT.

This meta-analysis demonstrates well the advantage of the network approach. Assessing the efficacy of treatments for Bell's palsy based on results of individual RCTs and direct meta-analysis is difficult due to the fact that there are various treatment regimens, and too few studies performing the same treatment comparisons for pooling. For instance, 6 treatment regimens are possible in clinical practice (i.e., Acyclovir, Valacyclovir, Prednisolone, Placebo, combination of Acyclovir + Prednisolone, and Valacyclovir + Prednisolone) resulting in 15 possible treatment-pair comparisons. Previous reviews have had problems with this multiplicity of comparisons:

• a previous systematic review of AVT versus corticosteroid[7] included only 3 studies with 246 patients, and these could not be pooled since each study had a different combination of treatments;

• one systematic review[34] included 3 studies with 117 patients and demonstrated no benefit of using corticosteroid compared with placebo/vitamin with relative risk of 0·86 (95% CI: 0·47 - 1·59).

• Another review[15] included 5 studies with 709 samples and reported no benefit of AVT (OR = 1·03; 95% CI: 0·74 - 1·42) when compared with Prednisolone.

The small numbers in these previous meta-analyses clearly led to lack of power. Two more complete reviews[14,17] were recently published. One accessed unpublished and non-English papers and thus included more studies than other previous meta-analyses. Point estimates of treatment effects for our results were similar to theirs, although confidence intervals varied. For instance, the effect of corticosteroid versus placebo at longer than 3 months was 0.54 in our study versus 0.69 for the recent meta but our study was slightly less precise (95% CIs were 0.40 - 0.74 versus 0.55 - 0.87, respectively). Effect of AVT plus corticosteroid versus AVT alone was also similar, i.e. the odds ratios for recovery were 0.49 (95% CI: 0.36 - 0.66) versus 0.48 (95% CI: 0.29 - 0.79). Our results are also consistent with the updated Cochrane review [17] which found a possibility of benefit of AVT plus corticosteroid versus corticosteroid which did not reach statistical significance, with the pooled point estimate of 1.41, and with the de Almeida et al. review which also found borderline evidence for a synergistic effect of steroids and AVT[14].

We have applied a mixed model for network meta-analysis[19,31]. The mixed model gains power by integrating both direct and indirect comparisons[15]. For instance, only two studies performed direct comparisons of Acyclovir plus Prednisolone versus Prednisolone alone, a total sample size of 409 (Table 2). The network method "borrowed" information on the Prednisolone group from three other studies and increased the total sample size to 773. Although our pooled estimates were quite heterogeneous, the mixed model with random intercept takes into account variation at the study level. In addition, goodness of fit assessment suggested that our model fit well with the data. Overall, our results are quite robust since discrepancies between the direct and the network analyses are small.

Quality of included studies varied; quality assessment scores ranged from 2-12. Meta-regression of direct meta-analysis indicated that this might be a source of heterogeneity in pooling effects of AVT. However, we could not adjust for the effects of quality assessment score and other co-variables in the mixed effect model since this requires individual patient data. An individual patient data meta-analysis could be attempted, although individual level raw data are often difficult and more time consuming to access. However, with this method, covariables in both study (e.g., quality assessment) and individual levels (e.g., age, disease severity) can be assessed using a multi-level analysis approach.

## Conclusion

Our evidence suggests that the current practice of treating Bell's palsy with AVT plus corticosteroid may lead to slightly higher recovery rates at 3 and 6 months compared to treating with corticosteroid alone, although at this point the sum of the data do not show that this is a significant difference; prednisone remains the best evidence-based treatment. Treating with AVT alone is significantly worse than treating with corticosteroid alone and is no better than placebo.

## List of abbreviations

AVT: Antiviral treatment; OR: Odds ratio; CI: Confidence interval; HSV: Herpes Simplex virus; NNT/NNH: Number needed to treat/harm

## Compteting interests

The authors declare that they have no completing interests

## Authors' contributions

AT had full accessed to all the data in the study and takes responsibility for the integrity of the data and the accuracy of the data analysis. Study concept and design: PN, AT, CD. Acquisition of data: PN, AT. Analysis and interpretation of data: PN, AT, JA. Drafting of the manuscript: PN, AT. Critical revision of the manuscript for important intellectual content: JA, CD. Final approval of the version to be published: all authors read and approved the final manuscript

Study supervision: AT, JA

## Pre-publication history

The pre-publication history for this paper can be accessed here:

http://www.biomedcentral.com/1471-2377/11/1/prepub

## Supplementary Material

Additional file 1 **Appendix**. Click here for file
