# Supplementary material for: Corticosteroid and antiviral therapy for Bell's palsy: A network meta-analysis
Source: BMC Neurol. 2011 Jan 5;11:1. doi: 10.1186/1471-2377-11-1 (PMC3025847; doi:10.1186/1471-2377-11-1)
Supplement: Additional file 1 — Appendix. [file 1471-2377-11-1-S1.DOC]

Appendix

1. Search strategies for MEDLINE

| **Query Number** | **Search Query** | **Number of Results** |
| --- | --- | --- |
| 1 | Facial nerve.mp. or exp Facial Nerve/ | 13645 |
| 2 | Paralysis.mp. or exp Paralysis/ | 73267 |
| 3 | 1 and 2 | 4306 |
| 4 | Facial Nerve Paraly$.mp. | 727 |
| 5 | exp Facial Paralysis/ | 8942 |
| 6 | Fac$ Paralys$.mp. | 9465 |
| 7 | Idiopath$.mp. | 64950 |
| 8 | (3 or 4 or 5 or 6) and 7 | 430 |
| 9 | Bell Palsy.mp. or exp Bell Palsy/ | 475 |
| 10 | Bell$ Pals$.mp. | 1619 |
| 11 | 8 or 9 or 10 | 1828 |
| 12 | Antiviral Agents.mp. or exp Antiviral Agents/ | 216400 |
| 13 | Antivir$.mp. | 59495 |
| 14 | Acyclovir.mp. or exp Acyclovir/ | 11784 |
| 15 | Valacyclovir.mp. | 758 |
| 16 | 11 and (12 or 13 or 14 or 15) | 149 |
| 17 | limit 16 to randomized controlled trial | 9 |

1. Search strategies for EMBASE

| **Query Number** | **Search Query** | **Number of Results** |
| --- | --- | --- |
| 1 | ‘facial nerve’/exp OR ‘facial nerve’ | 15,209 |
| 2 | ‘paralysis’/exp OR ‘paralysis’ | 113,809 |
| 3 | #1 and #2 | 8,611 |
| 4 | ‘facial nerve paralysis’/exp OR ‘facial nerve paralysis’ | 9,461 |
| 5 | ‘idiopathic’ | 59,501 |
| 6 | (#3 or #4) AND #5 | 494 |
| 7 | ‘bell palsy’/exp OR ‘bell palsy’ | 1,643 |
| 8 | bell* AND pals* | 2,119 |
| 9 | #6 OR #7 OR #8 | 2,321 |
| 10 | ‘antivirus agent’/exp OR antivir* | 353,107 |
| 11 | ‘aciclovir’/exp OR ac?clovir | 21,368 |
| 12 | ‘valaciclovir’/exp OR valac?clovir | 3,304 |
| 13 | #10 OR #11 OR #12 | 353,336 |
| 14 | #9 AND #13 | 260 |
| 15 | #14 AND [randomized controlled trial]/lim | 14 |
